# College and Hospital Notes

**Published:** 1910-12

**Authors:** 


### COLLEGE AND HOSPITAL NOTES

---

THE Buffalo General Hospital trustees held their annual meeting October 27, 1910, at which time Charles W. Pardee, Esq., was elected president for the seventh time. The other officers elected were: vice-president, Henry M. Watson; treasurer, Elliott C. McDougall; secretary, H. Edson Webster; trustees for three years, Frederick H. Stevens, Elliott C. McDougall, Robert C. Pomeroy, Charles W. Pardee, Dudley M. Irwin, Carlton M. Smith, Frank S. McGraw, Mrs. Truman G. Avery, Mrs. Bernard Bartow.

The new appointments to the staff are Dr. Irving P. Lyon, assistant attending physician; Dr. Francis C. Goldsborough, attending gynecologist and assistant attending obstetrician; assistant gynecologist, Dr. D. C. McKenney; assistants in obstetrics, Drs. W. T. Getman and F. H. Ransom.

---

DR. EDGAR F. SMITH, vice-provost of the University of Pennsylvania since 1898, was chosen provost of that institution November 15, 1910, to succeed Charles Curtis Harrison, whose resignation will become effective on December 31. Dr. Smith is a leading authority on electro-chemistry, and his work on that subject has been translated into several foreign languages, including Chinese. He is the author of numerous works on chemistry and is widely known for his researches in that field. He has taken a warm interest in all student activities, is extremely popular with graduates and undergraduates and is chairman of the faculty committee of athletics.

---

### To Examine Midwives.

COUNTY JUDGE TAYLOR has reappointed Dr. L. Kauffman, Dr. Frederick J. Parmenter and Dr. Nahum Kavinoky as members of the county board of midwifery examiners for a period of three years.

